# Comparison of markerless and marker-based motion capture systems using 95% functional limits of agreement in a linear mixed-effects modelling framework

**DOI:** 10.1038/s41598-023-49360-2

**Published:** 2023-12-18

**Authors:** Kishor Das, Thiago de Paula Oliveira, John Newell

**Affiliations:** 1 School of Mathematical and Statistical Sciences, University of Galway, Galway, Ireland; 2 CÚRAM, SFI Research Centre for Medical Devices, University of Galway, Galway, Ireland; 3 The Roslin Institute, University of Edinburgh, Edinburgh, Scotland; 4 The Insight Centre for Data Analytics, University of Galway, Galway, Ireland

**Keywords:** Biological techniques, Health care, Medical research, Mathematics and computing

## Abstract

Biomechanics analysis of human movement has been proven useful for maintenance of health, injury prevention, and rehabilitation in both sports and clinical populations. A marker-based motion capture system is considered the gold standard method of measurement for three dimensional kinematics measurements. However, the application of markers to anatomical bony points is a time consuming process and constrained by inter-, intra-tester and session reliability issues. The emergence of novel markerless motion capture systems without the use of reflective markers is a rapidly growing field in motion analysis. However an assessment of the level of agreement of a markerless system with an established gold standard marker-based system is needed to ensure the applicability of a markerless system. An extra layer of complexity is involved as the kinematics measurements are functional responses. In this paper a new approach is proposed to generate 95% functional limits of agreement (fLoA) using the linear mixed-effects modelling framework for hierarchical study designs. This approach is attractive as it will allow practitioners to extend their use of linear mixed models to assess agreement in method comparison studies in all domains where functional responses are recorded.

## Introduction

Biomechanics analysis of human movement has been proven to be invaluable for enhancing performance, maintenance of health, injury prevention, and aiding in rehabilitation in both sports and clinical population^1–6^. Specifically, three-dimensional kinematics data analysis can help early diagnosis of knee diseases, evaluating the effectiveness of medical surgery treatment, supporting clinical decision for surgeons and establishing rehabilitation programs in clinical settings^2,7–9^. In sport settings, kinematics data analysis facilitates the identification of athletes at elevated risk of injury and for designing athletic development programs as part of a long-term athletic development approach^1,4,5,10^.

Human movement analysis involves quantitative measurement of motion of a body joint during the execution of a locomotion and physical exercises with up to six degrees of freedom: three rotational components about the axes of a coordinate system and three translational components along these axes^11^. Usually, the Cartesian coordinate system is considered as the reference coordinate system which is considered to be embedded in the corresponding body joint. The reference coordinate system consists of three anatomical axes: anterior-posterior, medial-lateral, longitudinal axes, and corresponding three anatomical body planes: frontal, sagittal, and transverse planes^2^. The frontal plane divides the body into front and back parts, the sagittal plane divides the body into left and right sides and the transverse plane divides the body into top and bottom parts^2^. A motion in a particular plane occurs by rotation about an axis perpendicular to that plane. In particular, the anterior-posterior axis is perpendicular to the frontal plane, the medial-lateral axis is perpendicular to the sagittal plane, and the longitudinal axis is perpendicular to the transverse plane.

A motion that decreases joint angle in the sagittal plane is referred to as flexion, while a motion that increases the joint angle in the sagittal plane is referred to as extension^12^. Abduction refers to a motion away from the midline in the frontal plane, while adduction refers to the motion toward the midline. Joint motions in the transverse plane are referred to internal rotation and external rotation. These flexion/extension, abduction/adduction, and internal/external rotation motions are measured in angles which provide a quantitative description of a movement during a locomotion using three rotational degrees of freedom. These three sequences of angles along the three anatomical planes are the typical responses measured in studies collecting three-dimensional kinematics data. They can be viewed as a function of time and are therefore referred to as functional data in the literature^13^.

A marker-based motion capture system is considered the gold standard for three-dimensional kinematic measurements of joints^14^. This system, utilising reflective markers on anatomical landmarks according to the recommendations made by the International Society of Biomechanics (ISB), collects data on the trajectory of the reflective markers in space^11^. The trajectory of these markers are simultaneously captured by multiple cameras or optoelectronic sensors to determine the motion of a particular body segment in the three dimensional space^15^. Marker-based systems are considered the optimal laboratory based method to measure kinematics data with accuracy between 3${^\circ }$ and 5${^\circ }$ for most lower extremity segments^16,17^. However, there are several shortcomings of the marker-based systems^18^. First, there are many markers needed to be attached on the body which is a time consuming task and it has to be repeated for every new subject. Second, the system requires skilled personnel to apply the markers correctly which makes the system operator dependent. Third, the markers can influence the naturalness of the motion and subject to soft tissue artefact, the movement between the markers attached to the skin surface and the underlying bone causing inaccuracy in the measurements^19^. Finally, the system tends to be expensive compared to the markerless system^20^.

The emergence of novel markerless motion capture systems without the use of reflective markers is a rapidly growing field with an attractive future advancement in motion analysis^21,22^. A markerless motion capture system offers a fully automatic, non-invasive, markerless approach, which would ultimately provide a major breakthrough for research and practice within sports biomechanics and rehabilitation. This new technology uses multiple synchronised video cameras surrounding a capture space. If it is deemed reliable it could remove the difficulty of quantitatively assessing movement quality in elite academy footballers and ultimately have wider scope in scientific research. However, an assessment of the level of agreement of a markerless system with an established gold standard marker-based system is needed to ensure the applicability of a markerless system.

In this paper a novel approach for assessing the level of agreement between functional responses using 95% functional limits of agreement (fLoA) is proposed by extending the linear mixed-effects modelling (LMM) framework. The approach is demonstrated by analysing the agreement between functional responses recorded from a marker-based and a markerless motion capture system using a hierarchical study design. This approach is attractive as the fLoA provide an estimate of the bias and variance of the difference between two methods of measurement across the function in the actual unit of the observations and the LMM that these are based on can be easily adapted to accommodate the variety of study designs that an LMM can handle with the flexibility of adjusting for potential confounding covariates.

## Methods

### Study design

All of the nine participants were full time academy football players in an English Premier League club for over a minimum of 2 years and were all involved in supervised strength training. Participants had achieved full maturation status or 100% of peak adult height at the time of testing and were able to complete the lunge exercise without any physical restriction.

An experienced musculoskeletal physiotherapist with over 20 years of experience placed all the markers on the participants according to the guidance of an experienced biomechanists with over 15 years of motion capture experience. All methods were performed in accordance with the relevant guidelines and regulations of the International Society of Biomechanics (ISB)^23^. Ethics approval was obtained from the University of Galway Medical Ethics Committee. Individual informed consent obtained from each participant before testing. All participants were informed of the purpose of testing and advised that they could withdraw at any stage.

Kinematics data were collected using both the marker-based and markerless motion capture system simultaneously while all the participants performed the lunge exercise. All participants familiarised themselves with the movement prior to data collection under the supervision of a chartered physiotherapist and the same verbal and visual demonstration was used to instruct the participants in both testing occasions.

The time domain of the measurements for both systems were time normalised to 101 data points including the start and end of the exercise. This time domain is referred to as ‘normalised’ time or ‘time frame’ in this paper. The start of the lunge was defined as the first peak of right knee flexion as participants initially lifted their right leg up, and the end of the lunge was defined as the final peak in knee flexion as participants bend their knee prior returning to the start position.

For each performance of a lunge, three different joints were targeted for measurement: low spine, right hip, right knee. For each of the joints two motion capture systems were used simultaneously to measure three functional responses: flexion, abduction, and rotation angle curve. These functional responses are outcome measurements for this study. For each of the angle measurements, three replicate measurements were taken for each measurement session and there were two different measurement sessions. For this reason, the study design is hierarchical in nature. The details of the study and study design can be found elsewhere^24^.

### Motion capture systems

Measurements of lower extremity kinematics during a lunge were measured simultaneously by a marker-based and a markerless motion capture system. A brief descpription of the two systems are give here. More details of these systems can be found elsewhere^24,25^.

#### Marker-based

Eight infra-red cameras (Miqus, Qualisys Medical Ltd., Sweden) operating at 100 Hz surrounding the capture space were used to collect kinematic data for the marker-based system. According to the guideline of the manufacturer of the cameras, the capture volume was calibrated using the L-frame, a device provided by the manufacturer, which ensured maximum calibration residual of 1 mm for each camera. The 19 mm spherical reflective markers placed on the following sites: right and left anterior superior iliac spine, right and left posterior superior iliac spine, right and left medial femoral condyles, right and left lateral femoral condyles, right and left medial malleoli ankle, and right and left lateral malleoli ankle. Qualisys Track Manager$^{\tiny {\text {TM}}}$ (Version 2.16, Qualisys Medical Ltd., Sweden) was used to reconstruct the three-dimensional co-ordinates of each of the reflective markers. Rigid plates (131 $\times$ 80 mm) each consisting of four markers were strapped using Velcro tape to the left and right lateral thigh and shanks. The markers were placed width-wise 7cm apart and lengthwise 9 cm apart on the rigid plates. The two anterior markers of this rigid plate placed on the thighs and the medial and lateral knee markers were used to track the thighs^25^. Kinematic models were created using Visual 3D$^{\tiny {\text {TM}}}$ (version 6.01.16, C-motion, Germantown, MD, USA).

#### Markerless

Kinematic data were simultaneously captured using the markerless DARI system (Dynamic Athletic Research Institute, Motion Platform version 1.0.16-407 from Scientific Analytics Inc. Kansas City, KS, USA). The system is an eight camera based motion capture system operating at 50 Hz in the same dedicated motion capture space. According to the manufacturer’s instructions, the system was calibrated using the standard calibration board to achieve less than 3 mm reconstruction error.

#### Data pre-processing

The initial angle was subtracted from the whole curve for each of the responses. This new angle is known as “range of motion” in the biomechanics literature.

As the measurements of angles were taken using two different methods of measurement, the direction of the positive angle and negative angle were not similar for low spine body segments. For this reason all measurements of low spine angles by the markerless method were multiplied by $-1$ to align them and make them comparable to the direction of the positive angle of the marker-based method.

### Linear mixed-effects model

Linear mixed-effects models are used to model the relationship between a continuous response variable and a set of explanatory variables where the observations are grouped according to one or more categorical variables^26^. These models incorporate fixed effects, which are parameters associated with the entire population, and random effects, which are effects associated with individuals drawn at random from the population.

#### LMM for single level grouping

Consider $\varvec{y}_i$ as an $n_i$ dimensional response vector of observations for the *i*th group/subject. A mixed-effect model for this response can be written as:

1 $$\begin{aligned} \begin{aligned} \varvec{y}_i&= \varvec{X}_i \varvec{\beta } + \varvec{Z}_i \varvec{b}_i + \varvec{\varepsilon }_i, \quad i=1,\ldots ,n, \\&\varvec{b}_i \sim N(\varvec{0}, \varvec{\Psi }), \quad \varvec{\varepsilon _i} \sim N(\varvec{0}, \varvec{\Sigma }), \end{aligned} \end{aligned}$$

where *n* is the total number of groups, $\varvec{\beta }$ is a *p*-dimensional vector of fixed-effects, $\varvec{b_i}$ is a *q*-dimensional vector of random-effects, $\varvec{X}_i$ (of size $n_i \times p$) is the fixed-effects regressor matrix, $\varvec{Z}_i$ (of size $n_i \times q$) is the random-effects regressor matrix, and $\varvec{\varepsilon }_i$ is the $n_i$-dimensional error vector. Both the matrices $\varvec{\Psi }$ and $\varvec{\Sigma }$ are positive-definite symmetric. $\varvec{\Psi }$ is the variance-covariance matrix for the random effects that incorporates the correlation between the observations in the same group. $\varvec{\Sigma }$ is the variance-covariance matrix that incorporates the correlated and heteroscedastic residuals. The random-effects $\varvec{b}_i$ are assumed to be independent for different groups, the $\varvec{b}_i$ and the error $\varvec{\varepsilon }_i$ are independent within-group and between-groups.

#### Different random-effects structures

Different random-effects structures in the model (1) can be specified using a patterned variance-covariance matrix ($\varvec{\Psi }$) for the random-effects. If it can be assumed that random-effects are independent of each other, then the $\varvec{\Psi }$ matrix would be a diagonal matrix. When the random-effects are independent and have the same variance, then the $\varvec{\Psi }$ matrix would be an identity matrix multiplied by a constant. But if there is no structure assumed for the random-effects then the $\varvec{\Psi }$ matrix would be unstructured and for a $q\times q$ variance-covariance matrix for the random-effects $q\times (q+1) / 2$ parameters will be estimated in the $\varvec{\Psi }$ matrix.

#### Different error structures

A linear mixed-effects model often assumes that the within-group error is white noise. However, it may not be a suitable assumption to consider in many situations. In those situations, the within-group error could be *correlated* or *heteroscedastic* or both correlated and heteroscedastic. This correlated and heteroscedastic error is modelled using the covariance matrix $\varvec{\Sigma }$. This is done using the decomposition of the matrix $\varvec{\Sigma }$ into a product of matrices^26^

2 $$\begin{aligned} \varvec{\Sigma } = \varvec{V} \varvec{C} \varvec{V} \end{aligned}$$

where $\varvec{V}$ is a diagonal matrix and $\varvec{C}$ is the correlation matrix. To uniquely identify $\varvec{\Sigma }$ all the diagonal elements of $\varvec{V}$ must be positive^26^.

For a single level LMM, it can be shown that

3 $$\begin{aligned} \textrm{Var} (\varepsilon _{ij}) = [V]^2_{jj} , \quad \textrm{cor} (\varepsilon _{ij},\varepsilon _{ik}) = [C]_{jk} \end{aligned}$$

where $\varepsilon _{ij}$ is the error of the *j*th measurement from the *i*th subject and $\varepsilon _{ik}$ is the error for the *k*th measurement from the same subject. This decomposition of the covariance structure $\varvec{\Sigma }$ into a *variance structure* $\varvec{V}$ and a *correlation structure* $\varvec{C}$ is useful both theoretically and computationally. It allows one to model the two structures separately and then combine them in a more flexible framework. More details on different error structure can be found in the Appendix.

#### LMM for multi-level grouping

A single level LMM can be easily extended to a multi-level LMM. In this section, the single level LMM is extended into a two level LMM. For a two nested level LMM the response vector for the inner-most level can be written as $\varvec{y}_{ij}, i=1,\ldots ,n, j=1,\ldots ,n_i$, where *n* is the number of groups in the first level grouping, outermost, and $n_i$ is the number of second level groups within the first level *i*th group. The size of $\varvec{y}_{ij}$ is $n_{ij}$ and the LME model is^26^

4 $$\begin{aligned} \begin{aligned} \varvec{y}_{ij}&= \varvec{X}_{ij} \varvec{\beta } + \varvec{Z}_{i,j} \varvec{b}_i + \varvec{Z}_{ij} \varvec{b}_{ij} + \varvec{\varepsilon }_{ij}, \quad i=1,\ldots ,n,\quad j=1,\ldots ,n_i,\\&\varvec{b}_i \sim N(\varvec{0}, \varvec{\Psi }_1), \quad \varvec{b}_{ij} \sim N(\varvec{0}, \varvec{\Psi }_2), \quad \varvec{\varepsilon }_{ij} \sim N(\varvec{0},\varvec{\Sigma }), \end{aligned} \end{aligned}$$

where $\varvec{\beta }$ is the *p*-dimensional vector of fixed effects, $\varvec{b}_i$ is the $q_1$-dimensional vector of random effects for first level grouping, $\varvec{b}_{ij}$ is the $q_2$-dimensional vector of random effects for the second level grouping within the first level grouping, $\varvec{X}_{ij}$ (of size $n_{ij} \times p$) is the fixed effects regressor matrix, $\varvec{Z}_{i,j}$ (of size $n_{ij} \times q_1$) is the random effect regressor matrix corresponding to the $\varvec{b}_i,~\varvec{Z}_{ij}$ (of size $n_{ij} \times q_2$) is the random effect regressor matrix corresponding to the $\varvec{b}_{ij}, ~\varvec{\varepsilon }_{ij}$ is the error vector. $\varvec{\Psi }_1,~\varvec{\Psi }_2,~\varvec{\Sigma }$ are positive-definite symmetric matrices. The level-1 random-effects $\varvec{b}_i$ are assumed to be independent of different *i*, the level-2 random effect are assumed to be independent of different *i* or *j* and of $\varvec{b}_i$, the within-group errors $\varvec{\varepsilon }_{ij}$ are assumed to be independent of different *i* or *j* and of random effects.

#### Estimation for LMM

Maximum likelihood and restricted maximum likelihood estimation can be used to estimate the parameters in models (1). The details of the estimation procedure for both the ML and REML method and the discussion between the difference in the ML and REML method can be found elsewhere^26,27^. In this paper, only REML method will be used to estimate model parameters for a LMM as it incorporates the loss of degrees of freedom to estimate the fixed-effects parameters.

### Calculating 95% fLoA using a mixed-effects modelling framework

The idea for the methods presented in this section came from a paper where a nonparametric mixed-effects model for functional data combining B-spline basis functions was introduced within the existing mixed-effects modelling framework^28^. Their method proposed using B-spline basis functions for the fixed effects design matrix to model the nonlinear pattern in the mean curve and possibly a separate set of B-spline basis functions for the random-effects design matrix to model the covariance structure of the random-effects. The best linear unbiased prediction (BLUP) of the random effects can then be used to estimate the individual subject-specific curves.

Let $\varvec{y}_{mijk}$ be the vector of angle measured by the *m*th method ($m=m_{1}, m_{2}$), for the *i*th subject, *j*th session, and *k*th replicates. The frame-wise differences can be calculated as follows:

5 $$\begin{aligned} \varvec{d}_{ijk} = \varvec{y}_{m_{1}ijk} - \varvec{y}_{m_{2}ijk} \end{aligned}$$

where $\varvec{d}_{ijk}$ is the vector containing the difference between the measurements made by the two methods for the *i*th subject, *j*th session, and *k*th replicates. This $\varvec{d}_{ijk}$ will be the response vector for the LMM.

The model for this difference curve can be expressed as follows:

6 $$\begin{aligned} \begin{aligned} \varvec{d}_{ijk}&= \varvec{X}_{ijk} \varvec{\beta } + \varvec{Z}_{i,jk} \varvec{b}_{i} + \varvec{Z}_{ij,k} \varvec{b}_{ij} + \varvec{Z}_{ijk} \varvec{b}_{ijk} + \varvec{\varepsilon }_{ijk}, \\&i=1,\ldots ,9, \quad j=1,2, \quad k=1,\ldots ,4 \\&\varvec{b}_{i} \sim N(\varvec{0}, \varvec{\Psi }_{1}), \quad \varvec{b}_{ij} \sim N(\varvec{0}, \varvec{\Psi }_{2}), \quad \varvec{b}_{ijk} \sim N(\varvec{0}, \varvec{\Psi }_{3}), \quad \varvec{\varepsilon }_{ijk} \sim N(\varvec{0}, \varvec{\Sigma }) \end{aligned} \end{aligned}$$

where $\varvec{\Psi }_{1}, \varvec{\Psi }_2, \varvec{\Psi }_3, \varvec{\Sigma }$ are all positive-definite matrices. $\varvec{d}_{ijk}$ is a $n_i$-dimensional response vector containing the difference curve for a single replicate of a subject. $\varvec{X}_{ijk}$ is a $n_i \times p$ dimensional fixed-effects design matrix containing *p* B-spline basis functions, where the number *p* is sufficient to represent the mean difference curve. This number *p* depends on the number of knots and the degree of the spline. A cubic spline with a suitable knot sequence will be chosen for the B-spline basis. $\varvec{\beta }$ is a *p*-dimensional vector containing the fixed-effects corresponding to each column of the matrix $\varvec{X}_{ijk}$.

$\varvec{Z}_{i,jk}$ is a $n_i \times q_1$ dimensional random-effects design matrix containing $q_1$ B-spline basis functions. This matrix models the random deviation of the individual subject from the mean curve. $\varvec{b}_{i}$ is a $q_1$-dimensional vector containing the random-effects corresponding to the matrix $\varvec{Z}_{i,jk}$.

$\varvec{b}_{ij}$ is a $q_2$-dimensional vector containing random-effects for the *j*th session for a given subject $i. \, \varvec{Z}_{ij,k}$ is a $n_i \times q_2$ dimensional matrix containing $q_2$ B-spline basis functions. $\varvec{b}_{ijk}$ is a $q_3$-dimensional vector containing the random-effects for the *k*th replicates for a given subject *i* and a given session *j*. $\varvec{Z}_{ijk}$ is the corresponding random-effect design matrix which is $n_i \times q_3$ dimensional. $\varvec{\varepsilon }_{ijk}$ is the $n_i$-dimensional error vector.

The level-1 random effects $\varvec{b}_{i}$ are assumed to be independent for different *i*, the level-2 random effects $\varvec{b}_{ij}$ are assumed to be independent for different *i* or *j* and independent of level-1 random effects, the level-3 random effects $\varvec{b}_{ijk}$ are assumed to be independent of *i*, *j* or *k* and independent of level-1 and 2 random effects, the within-group errors $\varvec{\varepsilon }_{ijk}$ are assumed to be independent of different *i*, *j* or *k* and independent of all the level-1,2,3 random effects.

The bias between the two methods of measurement is

7 $$\begin{aligned} \varvec{\mu }_d = \textrm{E}(\varvec{d}) = \varvec{X}_{ijk} \varvec{\beta } \end{aligned}$$

and the variance-covariance matrix of the differences between pairs of measurements is

8 $$\begin{aligned} \begin{aligned} \textrm{Var}(\varvec{d})&= \textrm{Var}\Big [ \varvec{X}_{ijk} \varvec{\beta } + \varvec{Z}_{i,jk} \varvec{b}_{i} + \varvec{Z}_{ij,k} \varvec{b}_{ij} + \varvec{Z}_{ijk} \varvec{b}_{ijk} + \varvec{\varepsilon }_{ijk}\Big ]\\&= \varvec{Z}_{i,jk} \varvec{\Psi _{1}} \varvec{Z}_{i,jk}' + \varvec{Z}_{ij,k} \varvec{\Psi _{2}} \varvec{Z}_{ij,k}'+ \varvec{Z}_{ijk} \varvec{\Psi _{3}} \varvec{Z}_{ijk}'+\varvec{\Sigma }\\&= \varvec{\Lambda }_d \end{aligned} \end{aligned}$$

The 95% limits of agreement for the functional response are

9 $$\begin{aligned} \varvec{\mu }_d \pm 2 \, \sqrt{diag(\varvec{\Lambda }_d)} \end{aligned}$$

To calculate the 95% fLoA for functional responses, model (6) needs to be fitted using the restricted maximum likelihood estimation procedure^26^. In order to fully specify the mixed-effects model, three key decisions are necessary. First, an appropriate knot sequence for a B-spline basis system for the fixed-effects regressor matrix must be determined. This knot sequence will significantly affect the fit of the model to the data and the interpretation of the fixed effects. Next, one needs to select the knot sequence for a B-spline basis system for the random effects regressor matrix and then specify the variance-covariance matrix for the random effects. This step is crucial because it determines how the random effects, which capture subject-specific deviations from the fixed effects structure, are allowed to co-vary. The final decision concerns the selection of an appropriate correlation structure for the error. This structure captures the correlation of the residuals and can significantly impact the model’s goodness of fit and the accuracy of inferences. It is important to choose a correlation structure that accurately reflects the structure of the data to avoid incorrect inferences.

#### Fixed-effects structure

To model the mean curve, a choice of a set of B-spline basis functions for the fixed-effects regressor matrix is needed. This depends on the degree of the spline curve and the position of the knot sequence. As derivatives of the curves are not of interest for the method comparison study, a cubic spline will be sufficient^13^ and only a sequence of inner knots needs to be selected. However, if one wishes then it is also possible to choose a B-spline basis system that allows higher order derivatives of curves to be estimated^29^. There are many possible choices to consider for the knot sequence. Figure 2 shows four different options to choose from for the sequence of knots for the fixed effect structure. These are not the only possible choices, but when there is no reason to put knots on specific points on the domain, an equally spaced knot sequence seems a reasonable choice. In Fig. 2, there are no inner knots for the spline functions in the top left panel. There is only one inner knot for the top right panel in the middle of the domain. There are three and nine equally spaced inner knots in the bottom two panels. Depending on the number of inner knots, a different B-spline basis system will be generated with a different number of basis functions. An objective criterion is needed to pick a suitable combination of these for the context in question, in this case a motion capture study.

One way of selecting a knot sequence is to fit different LMMs with different knot sequences and use the Akaike information criterion (AIC) or Bayesian information criterion (BIC) to choose one. However, this would be a cumbersome task as there are many possible choices for the sequence of knots. In this paper, a more straightforward approach is proposed to guide the choice of the appropriate knot positions. The approach is based on the adjusted R-squared statistic. Separate regression splines will be fitted for each of the individual replicate measurements for different sets of B-spline basis systems. Finally, a sequence of knots will be chosen where all of the adjusted R-squared values are 0.95 or more.

#### Random-effects structure

To choose a suitable basis system for the random-effects regressor matrix one can fit different LMMs with a different B-spline basis system for the random-effects design matrix and then choose a suitable one based on the AIC or BIC^28^. However, this would also be cumbersome task as many models must be fitted in order to choose an appropriate basis system for the random-effects regressor matrices. In this paper a more straightforward approach was considered, that is to choose the basis system for the random-effects structure same as the fixed-effects structure.

In addition to the specification of the B-spline basis system, the structure of the variance-covariance matrix must be specified. Here an unstructured variance-covariance matrix for the random-effects will be considered as it has been found that an unstructured variance-covariance matrix for the random-effects is essential to correctly estimate the variance curve^30^.

#### Correlation structure for error

Choosing the most appropriate correlation structure for the error of the LMM to calculate 95% fLoA is the next consideration. The list of correlation structures and the corresponding R function in the nlme package can be found elsewhere^26^.

The strategy is to fit different LMMs with different correlation structures by looking at the autocorrelation plot for each model and then deciding which correlation structure best removes the serial correlation from the residuals.

#### Guideline to choose different model components

The various choices needed to generate 95% fLoA will clearly differ from context to context. That said, the overall guidelines below can be used as a sensible strategy to employ when using a LMM to calculate 95% fLoA. These are as follows:

- For the fixed-effects regressor matrix, choose a cubic B-spline basis system with a knot sequence suggested by the adjusted R-squared criterion.
- Choose the same B-spline basis system for the random-effects regressor matrix.
- The variance-covariance matrix for the random-effects must be unstructured.
- Choose an appropriate correlation structure for the error structure based on the autocorrelation plot.

#### Faster computational approach for nonparametric LMM

In this section, a novel faster computational approach is proposed to fit a non-parametric LMM to calculate 95% fLoA using a LMM with a modified basis system for the random-effects regressor matrix which allows a diagonal variance-covariance matrix for the random-effects to be implemented. The approach is based on the results of the Karhunen-Loève theorem which will now be introduced.

Let $X_t$ be a zero-mean and square-integrable stochastic process defined over some probability space with continuous covariance function $K_{X}(s, t)$. $X_t$ is defined over a closed interval [*a*, *b*]. A linear operator $T_{K_X}$ can be defined as follows:

10 $$\begin{aligned} T_{K_X} f = \int ^a_b K_X(t,.) f(t) dt \end{aligned}$$

with *k*th eigenvalue $\lambda _k$ and corresponding eigenfunction $e_k$^31^. According to the Karhunen-Loève theorem, the stochastic process $X_t$ can be represented as follows^31^:

11 $$\begin{aligned} X_{t} = \sum ^{\infty }_{k=1} c_k e_k(t) \end{aligned}$$

Here the coefficient $c_k$ is a random variable with mean zero and variance $\lambda _k$ with the following definition^31^:

12 $$\begin{aligned} c_k = \int ^a_b X_t e_k(t) dt \end{aligned}$$

Consider the functional response $\varvec{y}$ as a stochastic process where values were obtained at certain equally spaced time points. $\varvec{y}$ can be modelled as follows:

13 $$\begin{aligned} \varvec{y} = \varvec{X} \varvec{\beta } + \varvec{Z} \varvec{b} + \varvec{\varepsilon } \end{aligned}$$

where the mean of $\varvec{y}$ is $\varvec{X} \varvec{\beta }$ with covariance matrix $\varvec{\Sigma }$. Then $\varvec{y} -\varvec{X} \varvec{\beta }$ is a zero-mean stochastic process with covariance matrix $\varvec{\Sigma }$. Since

14 $$\begin{aligned} \varvec{y} - \varvec{X} \varvec{\beta } = \varvec{Z} \varvec{b} + \varvec{\varepsilon } \end{aligned}$$

$\varvec{Z} \varvec{b} + \varvec{\varepsilon }$ is also a zero-mean stochastic process with covariance matrix $\varvec{\Sigma }$. Therefore, $\varvec{Z} \varvec{b}$ is a zero-mean stochastic process with covariance matrix different to $\varvec{\Sigma }$. Consider,

15 $$\begin{aligned} \varvec{u} = \varvec{Z} \varvec{b} \end{aligned}$$

On comparing Eq. (11) with Eq. (15) it is clear that the column of the $\varvec{Z}$ can be the eigenvectors of the $\varvec{\Sigma }$ and $\varvec{b}$ a vector of independent random coefficients of the corresponding eigenvectors. Although $\varvec{\Sigma }$ is not the covariance matrix of $\varvec{u}$, it does not matter as the coefficients will not be estimated by Eq. (12). Only a convenient basis system for $\varvec{u}$ is needed, and the REML criterion with BLUP will provide estimates of the coefficients of the random-effects $\varvec{b}$.

Let $\varvec{S}$ be the sample covariance matrix for a sample of *n* curves. These *n* curves are *n* realisation of the random vectors $\varvec{y}$. Each curve consists of $n_i$ equally spaced observations. The sample covariance matrix is an $n_i\times n_i$-dimensional matrix. The eigenvalue decomposition of the matrix $\varvec{S}$ is as follows:

16 $$\begin{aligned} \varvec{S} = \varvec{Q}\varvec{\Lambda }\varvec{Q'} \end{aligned}$$

where $\varvec{\Lambda }$ is a $n_i\times n_i$ diagonal matrix with eigenvalues as diagonal elements, $\varvec{Q}$ is a $n_i\times n_i$ orthogonal matrix where each column contains an eigenvector. The *i*th column of the $\varvec{Q}$ matrix is the eigenvector associated with the $[\varvec{\Lambda }]_{ii}$ eigenvalues. After this decomposition, one can look at the number of non-zero eigenvalues. The eigenvectors associated with those non-zero eigenvalues would then be the set of basis functions or vectors in the $\varvec{Z}$ matrix in the Eq. (13). In practice, not all the eigenvectors will be considered. Eigenvectors corresponding to the smallest eigenvalues can be ignored. This representation provides a basis system for the random-effects design matrix where a diagonal covariance structure for the random-effects can be assumed as the eigenvectors are orthogonal to each other.

In situations where a large number of B-spline basis functions are required for the random-effects regressor matrix, the optimisation process can become time-consuming. In such instances, adopting an approach that uses an eigenbasis for the random-effects regressor matrix, paired with a diagonal variance-covariance matrix for the random effects, can prove highly beneficial. This strategy helps to streamline the computational process, offering a more efficient solution.

A recommendation on how to specify an LMM when computation time is an issue to calculate the 95% fLoA using an eigenbasis is as follows:

- For the fixed-effects regressor matrix, choose a cubic B-spline basis system with a knot sequence suggested by the adjusted R-squared criterion.
- Calculate the variance-covariance matrix for the functional responses and obtain an eigenvalue-eigenvector decomposition of the matrix.
- Choose the first few eigenvectors that explain at least 99% of the variation of the responses.
- Use a diagonal variance-covariance matrix for the random-effects.
- Choose a appropriate correlation structure for the error based on the autocorrelation plot.

## Results

**Figure 1:**
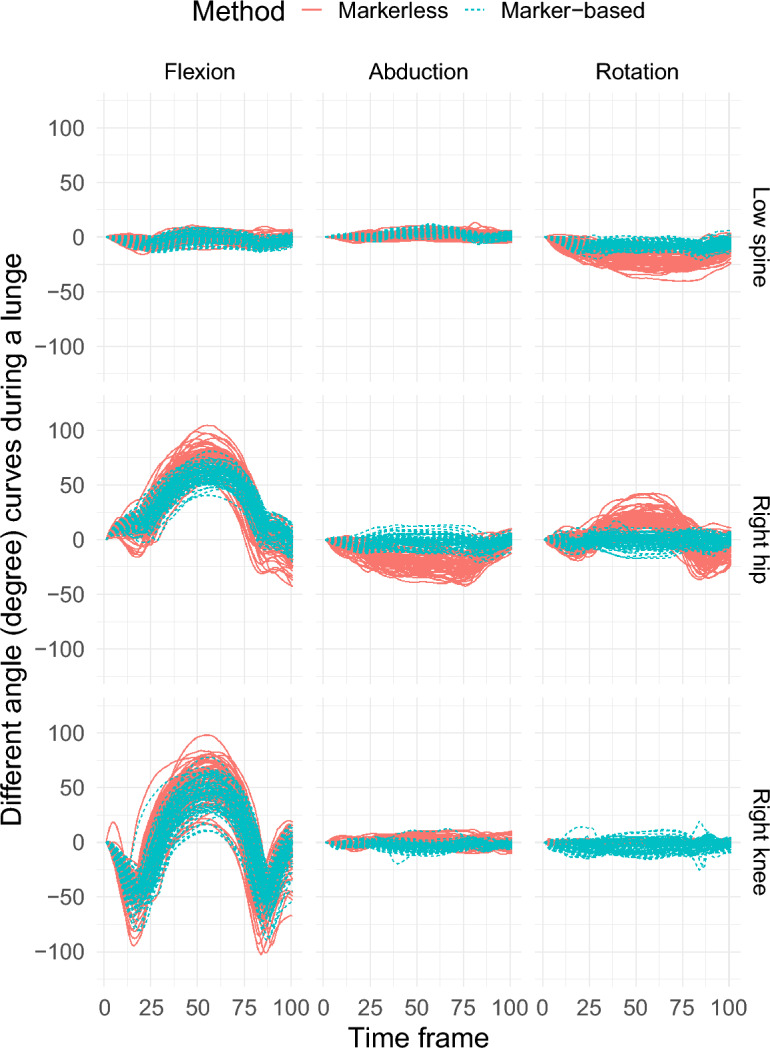
Shape of the different angle curves in different anatomical planes at different body segments measured by two different methods of measurement during a right leg lunge.

Nine elite soccer players (n=9) with mean (SD) age 18.5 (1.3) years, height 1.83 (0.04) metres and weight 79.2 (6.2) kg participated in the study. For the right leg lunge exercise three joints were considered, i.e. low spine, right hip and right knee. For each joint, three different angle curves were measured to capture the flexion/extension, abduction/adduction, and internal/external rotation movement. This yields nine different scenarios to compare the two motion capture systems. Figure 1 displays the functional responses collected for all the nine different scenarios using the two motion capture systems. For each scenario (e.g. low spine flexion angle) 108 functional responses were measured. These functional responses consist of measurements obtained by the two motion capture systems from nine different subjects in two measurement sessions and three replicates for each session. The shape of the functional responses and their variability over time are clearly different for each scenarios (Fig. 1). It can be observed that the variability of the markerless system is higher compared to the marker-based system. It appears that the level of agreement between the two methods of measurement is not the same for each scenario. For example, agreement between the methods of measurement for a right hip flexion angle seems better compared to the agreement for a right hip rotation. Hence, the assessment of agreement between the two methods of measurement will be investigated separately for each of scenarios.

To assess the agreement between the two motion capture systems, frame-wise differences were calculated first. After calculating the difference curves, each scenario has 54 difference curves. These difference curves were then modelled using a non-parametric linear mixed-effects model (LMM). Then 95% fLoA were calculated using the estimated model parameters of the fitted LMM. Since the shape and variability of functional responses are different in different scenarios, nine LMMs were fitted for the nine different scenarios.

The procedure of calculation of the 95% fLoA using a non-parametric LMM will first be demonstrated using one scenario, i.e. right hip abduction angle. The same procedure will then be used to calculate 95% fLoA for all other scenarios. Fitting a non-parametric LMM requires specification of the fixed-effects regressor matrix, random-effects regressor matrix, variance-covariance matrix of the random-effects, and error structure.

**Figure 2:**
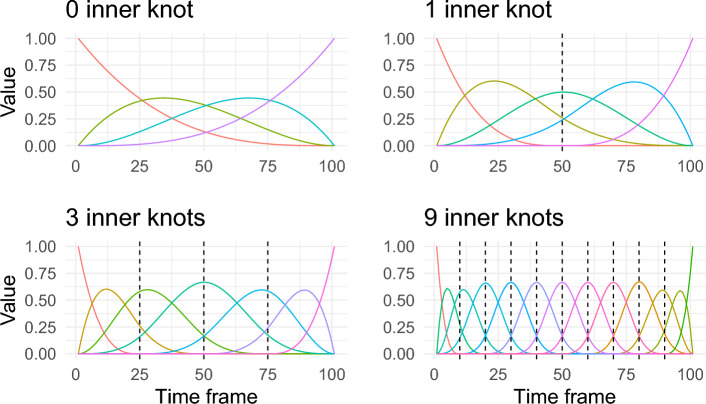
Cubic B-spline basis functions with a different number of equally spaced inner knots.

To model the mean curve, a choice of a set of B-spline basis functions for the fixed-effects regressor matrix is needed. There are many possible choices to consider for the knot sequence. Figure 2 displays four different options to choose from for the sequence of knots for the fixed-effect structure in question. These are not the only possible choices, but when there is no reason to put knots on specific points on the domain, equally spaced knot sequence seems a reasonable choice. In Fig. 2, there is no inner knot for the spline functions in the top left panel, only one inner knot in the middle of the domain for the top right panel and three and nine equally spaced inner knots respectively in the bottom two panels. Depending on the number of inner knots, a different B-spline basis system will be generated with a different number of basis functions.

**Figure 3:**
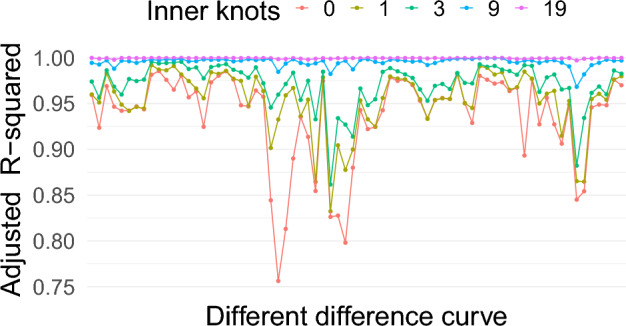
Adjusted R-squared values after fitting regression splines to each individual difference curve using B-spline basis functions with different numbers of inner knots as the covariates. Here points on the x-axis represent different difference curves. In total 54 difference curves (9 subjects, 2 sessions, 3 replicates) were used for the right hip abduction angle measured by two different measurement methods: markerless and marker-based.

Figure 3 shows the adjusted R-squared statistics calculated from the individual difference curves after fitting a regression spline to each curve using the set of basis functions as the covariates. It suggests that the sequence of knot positions with zero inner knots cannot model all the individual curves adequately. As one increases the number of inner knots, the set of basis functions can model more individual curves. For example, with 19 inner knots, the model can accommodate all the individual curves very well; however, with only nine inner knots, the fit is almost as satisfactory as the fit with 19 inner knots. For this reason, a parsimonious B-spline basis system with 9 inner knots will be considered for the fixed-effects regressor matrix. This provides 12 basis functions for the fixed-effects regressor matrix for the right hip abduction angle curves.

The B-spline basis system chosen for the fixed-effect matrix can model individual difference curves sufficiently well. Hence, this basis system can also be sufficient to model subject-specific deviation curves from the mean curve. For this reason the same B-spline basis system will be used for the random-effects regressor matrix. Note that due to the hierarchical nature of the study design, three different random-effects regressor matrices will be needed given the levels of the hierarchy, i.e. subject, session, and replicate level. For each of the three levels the same B-spline basis system will be used. Unstructured variance-covariance matrices are essential for the random-effects at different levels. Since a random-effects regressor matrix at each level contains 12 basis functions, the corresponding variance-covariance matrix requires 78 $(12\times 13/2)$ parameters to be estimated. In total 234 $(78\times 3)$ variance parameters will be needed to be estimated. This would be a very computationally expensive estimation procedure. For this reason an eigenbasis will be used for the random-effects regressor matrices to achieve computationally faster estimation. It has been found that 12 eigenbasis will be sufficient for the random-effects regressor matrices for the right hip abduction difference curves. This allows one to consider only diagonal variance-covariance matrices for the random-effects. The computation time to fit the LMM with enigenbasis including the error structure took about 16 min whereas the LMM with the full B-spline implementation excluding the error structure took about 52 min.

**Figure 4:**
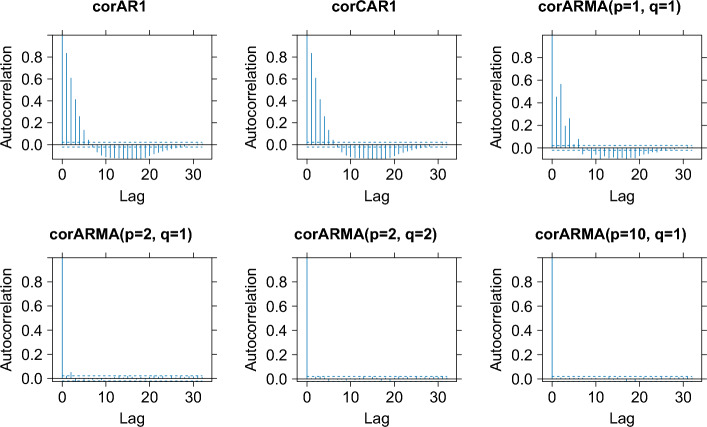
Auto-correlation plots of the residuals after fitting a mixed-effects model with different temporal correlation structures for the right hip abduction angle data. Here, AR1 is an autoregressive model with order 1; CAR1 is a continuous autoregressive model with order 1; ARMA(p, q) is an autoregressive-moving-average model with order p for the autoregressive model and order q for the moving-average model.

After choosing the basis system for the fixed-effects and random-effects regressor matrices, a suitable error structure must be specified. As the data are time series in nature, an autoregressive-moving average (ARMA) model for the error is the most natural choice. Figure 4 shows the autocorrelation plot after fitting different LMMs with different ARMA models for the error. It shows that an ARMA with order 2 for the autoregressive process and order 1 for the moving average process removed the autocorrelation from the residuals.

**Figure 5:**
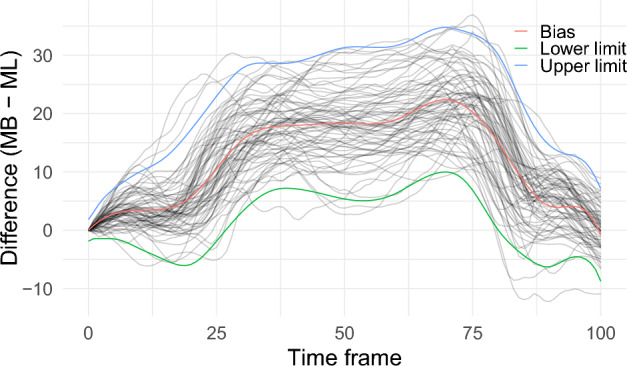
95% functional limits of agreement estimated by a linear mixed-effects modelling framework with an eigenbasis systems for the random-effects regressor matrices; MB is the marker-based and ML the markerless method of measurement.

After fitting an LMM to the difference curves obtained for the right hip abduction angle, the 95% fLoA were calculated (Fig. 5). In Fig. 5, the grey lines are the difference curves produced by the pair of measurements by the two methods of measurement. The red curve is the relative bias curve. The blue curve is the upper limits of agreement band and the green curve is the corresponding lower limits of agreement. The 95% fLoA should be interpreted as pointwise limits. This means that for a given time point the upper and lower limits of agreement represent the range that contain at least 95% of the differences in values at that frame.

For the bias curve it can be observed that there is substantial bias between the two methods of measurement from frames 25 to 75. It can be observed that the markerless method overestimates measurements by 18${^\circ }$ to 22${^\circ }$ on average. This means that, on average, the markerless methods overestimated the abduction/adduction angle by 18${^\circ }$ to 22${^\circ }$ for the mid part of the domain during a lunge. Limits of agreement indicates that for individual measurement, it is likely that the markerless method can overestimate measurements from 5 to 30 units which are considerably large deviations. From this it can be concluded that the two methods of measurement do not agree when measuring right hip abduction angle during a right leg lunge.

**Figure 6:**
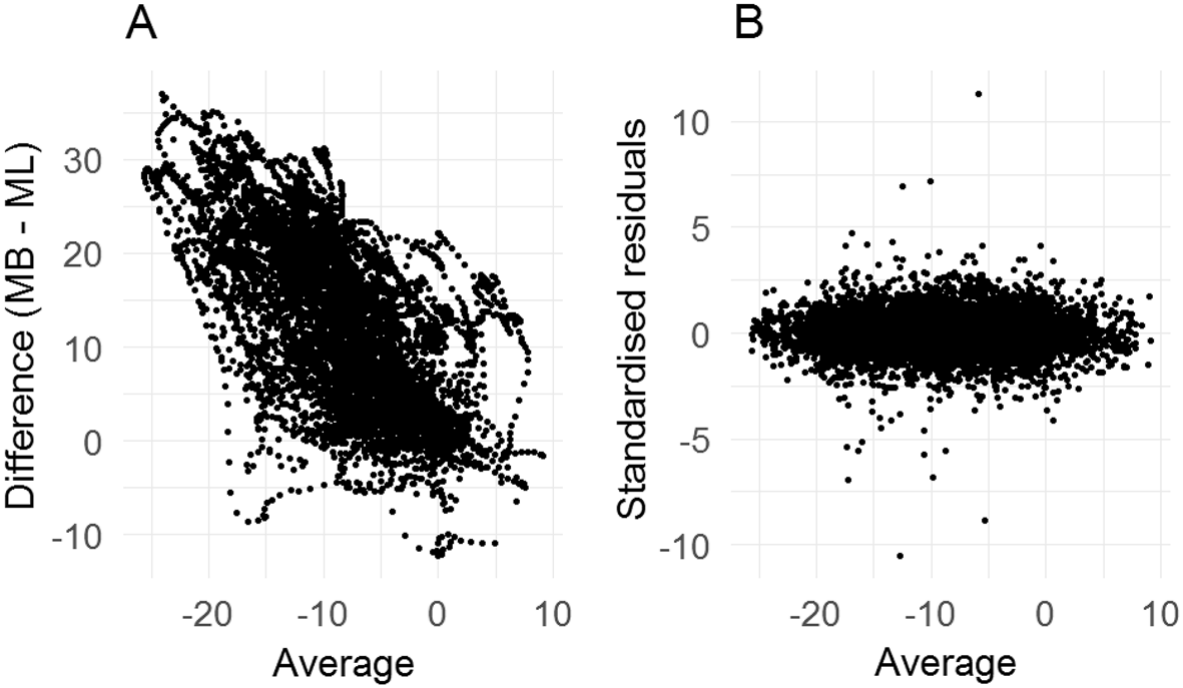
(**A**) Differences against averages ignoring the time frame for the right hip abduction angle curves. All the replicate measurements from two measurement sessions were considered here. (**B**) Residuals against averages for the same data after fitting a linear mixed-effects model with eigenbasis for the random-effects regressor matrices; MB is the marker-based and ML is the markerless method of measurement.

In order to interpret 95% LoA correctly, there is an assumption that the mean and standard deviation of the difference should be constant over the range of measurements. This assumption can be checked using the so called Bland-Altman plot^32^. However, when a modelling framework is used this assumption does not need to be satisfied as the non-linear pattern of the bias and variability of the measurement can be modelled using the modelling framework. The corresponding assumption is to check if the residuals have any pattern of concern when plotted against the average value ignoring the time dimension (Fig. 6). The average values here represent the range of measurements. When there is no pattern in the residuals while plotted against the range of measurements, it can be stated that the modelling framework has been successful to model the non-linearity in the bias and variance in the data. Figure 6A is a plot of the difference against the average ignoring the time dimension. Here one can see that there is a pattern in the differences when plotted against the averages which indicates that a modelling framework is need to model this non-linearity. Figure 6B is a plot of the residuals obtained from the model against the average values ignoring the time dimension. Here it seems that there is not any pattern in the residuals after fitting the model. This ensures that the model is appropriate to calculate the 95% fLoA.

**Figure 7:**
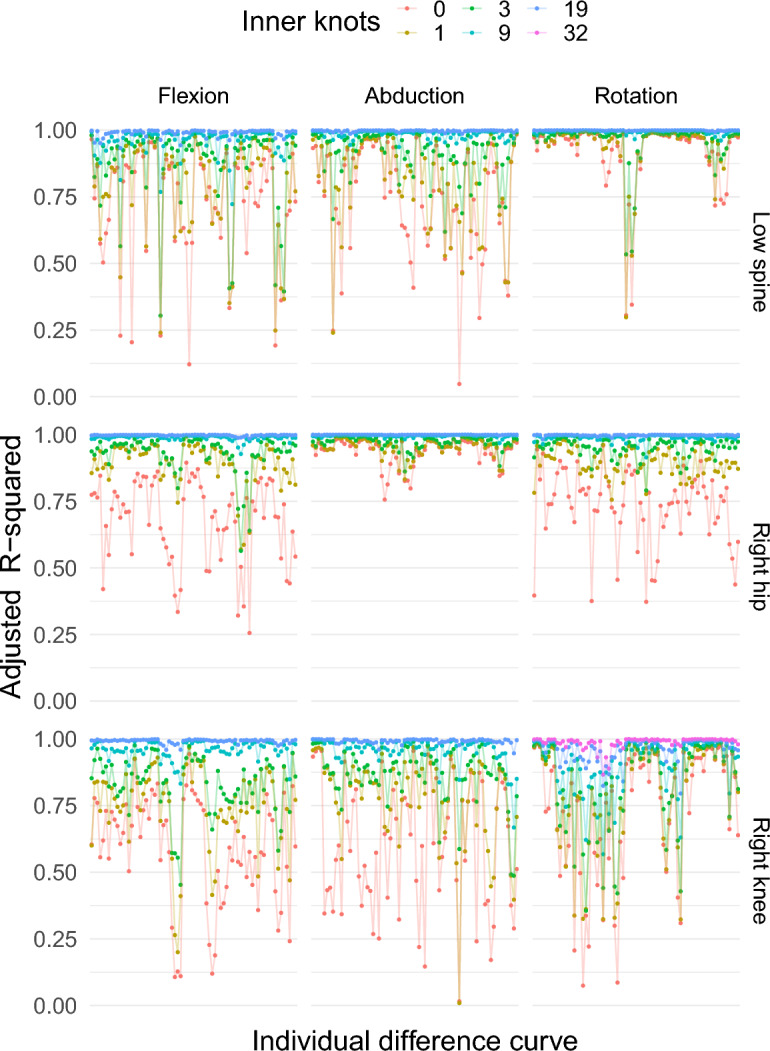
Adjusted R-squared values after fitting a regression spline to each individual difference curve using B-spline basis functions with different numbers of equally spaced inner knots for different angles measured in the motion capture study. The different points on the x-axis represent different difference curves. For each angle, 54 difference curves (9 subjects, 2 sessions, 3 replicates) were calculated for the markerless and marker-based systems.

Calculation of the 95% fLoA using the LMM framework has been demonstrated for the right hip abduction angle. The same framework will be used to calculate the 95% fLoA for all the other scenarios. As the shape of the functional responses are different for each scenario, different knots sequences are needed. A criterion based on the adjusted R-squared was used to choose the sequence of knots for a B-spline system, as demonstrated before. Figure 7 shows values of the adjusted R-squared after fitting a regression spline for each individual difference curve for all the scenarios. Table 1 shows the number of inner knots needed for the knot sequence of the B-spline basis system for the fixed-effects regressor matrix of the LMM for different scenarios.

**Table 1:** LMM specification for the difference curves by angles with the time taken to fit the model.

Angles	Fixed-effects (inner knots)	Random-effects (eigenfunctions)	Error structure	Time to fit (in minutes)
Low spine flexion	19	12	ARMA(2,1)	22.92
Right hip flexion	9	12	ARMA(2,1)	20.14
Right knee flexion	19	15	ARMA(2,1)	31.45
Low spine abduction	19	12	ARMA(2,1)	27.35
Right hip abduction	9	12	ARMA(2,1)	18.86
Right knee abduction	19	14	ARMA(2,1)	34.10
Low spine rotation	9	12	ARMA(2,1)	20.10
Right hip rotation	9	12	ARMA(2,1)	21.93
Right knee rotation	32	14	ARMA(2,1)	31.68

After choosing the basis system for the fixed-effect regressor matrix, the same basis system as for the fixed-effects regressor matrix can be used for the random-effects regressor matrix. However, in this case, an unstructured variance-covariance for the random-effects is necessary. For this reason an eigenbasis system is used so that a diagonal variance-covariance matrix can be considered for the random-effects. Table 1 shows the different number of eigenfunctions needed for the random-effects regressor matrix for the different scenarios considered.

**Figure 8:**
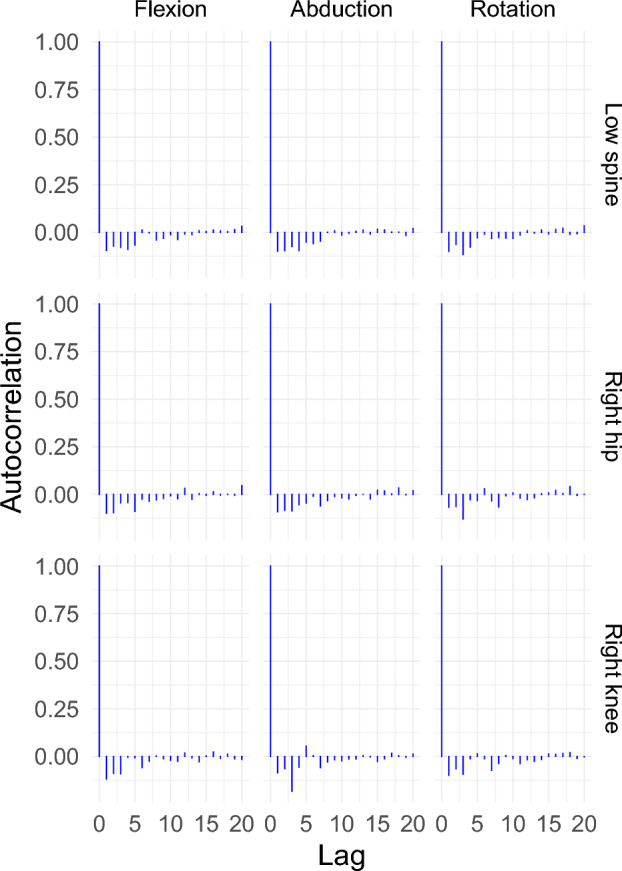
Autocorrelation plot of the residuals after fitting a LMM of the difference curves with an autoregressive moving average ARMA(2,1) error structure for the lunge angles.

After choosing basis systems for both the fixed-effects and random-effects regressor matrices, the next step is to specify a suitable correlation structure for the error. Once again an autoregressive-moving average (ARMA) is a natural choice for this situation where an ARMA(2, 1) was used for the error in the LMM in this instance. Figure 8 shows that the error structure is sufficient to remove any correlation in the residuals after fitting an LMM for each of the scenarios.

**Figure 9:**
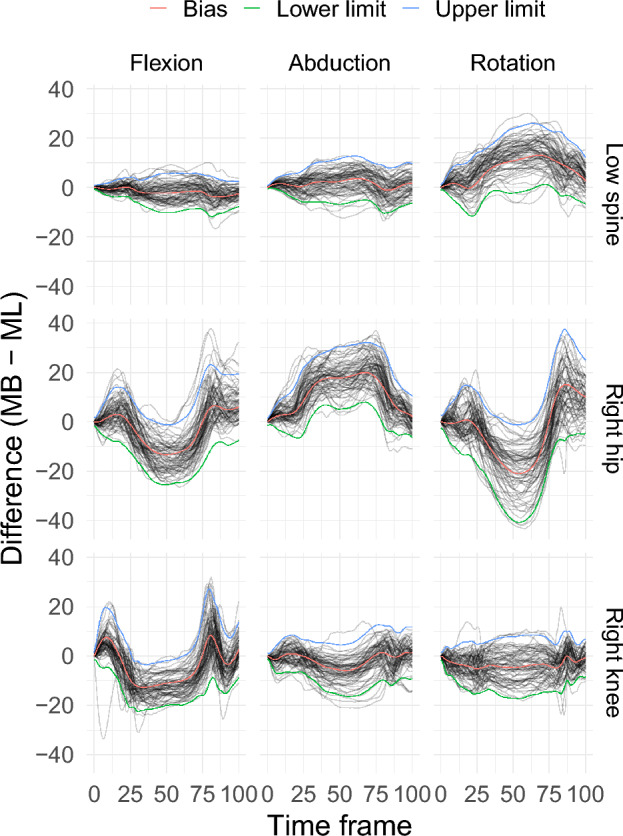
95% functional limits of agreement using a linear mixed-effects model for all angles measured during a lunge. Here MB is the marker-based and ML is the markerless method of measurement.

These model specifications were used to fit a LMM for each of the scenarios. Using the model, the 95% fLoA were calculated to assess the agreement between the two motion capture systems for each different scenario (Fig. 9).

## Discussion

Limits of agreement have been the preferred statistical method to analyse method agreement studies for univariate responses. To extend the 95% LoA developed for univariate responses, an extension was proposed in the literature based on the functional data analysis^33^. The methodology is only applicable for studies without any functional replicates. To extend the 95% LoA for a study with functional replicates, an approach has been proposed using a mixed effect modelling approach^34^. In this approach 100 different mixed effect models were fitted, one for each time point, to estimate the 95% confidence band for mean difference curve. This 95% confidence band is not a proper extension of the 95% LoA for curve data. In addition, to fit 100 different mixed effect models to produce the mean difference curve and corresponding confidence band is a cumbersome approach to analyse data. In this paper, a novel approach has been proposed to calculate 95% fLoA for functional responses using a non-parametric mixed-effects modelling framework which allows method comparison studies to be analysed involving functional responses from study designs that an LMM can handle. Fitting a non-parametric mixed-effects model is time consuming process. A novel computational approach is also proposed to fit a non-parametric LMM for functional responses.

One of the main contributions of this paper therefore is to present a unified and computationally efficient approach for generating functional limits of agreement through a mixed-effects modelling framework.

A distance-based quantity, e.g. dynamic time warping (DTW), can be used to compare time series data, hence can be used to compare two methods of measurement. However, the variation between these distances is not easy to interpret. The method proposed in this paper is attractive compared to any distance-based comparison methods as it can provide an estimate of the bias curve and the variation of the difference curves in the original units of measurement. This bias can be used as an offset to post-process measurements taken using the new method. In addition to that different scientific disciplines may require different level of precision of the method of measurement. For example, accuracy of the device to measure inflammation in competitive sports may be different compared to medical practice. For this reason, one device might be useful in a particular setting but not in other settings. The variation of the differences can be used to quantify the precision of the measurements to decide whether it is applicable to a particular setting or not.

Kinematic data of the lower limb were collected simultaneously using both a marker-based and markerless system during a lunge. It can be observed that the variability of the curves for the markerless system is greater compared to the variability of the marker-based system (Fig. 1). This means that the markerless system is less reliable compared to the marker-based system for the given contexts explored in this paper. The level of agreement between the two systems was assessed in nine different scenarios, i.e. low spine, right hip and right knee angle in sagittal, frontal and transverse plane. 95% fLoA were calculated for each of the nine different scenarios to assess the agreement between the two methods.

To assess the agreement between the two systems for each scenario, two aspects of the 95% fLoA should be considered, i.e. the bias curve and the width of the band produced by upper and lower limits of agreement. The bias curve indicates how good the agreement is between the two systems on average. If there is perfect agreement between the two systems the bias curve should be a horizontal zero line. For the low spine angle, the bias between the two systems is negligible in the sagittal and frontal plane, however the bias could be as high as 10${^\circ }$ to 13${^\circ }$ on average in the transverse plane. For the right hip angle there is substantial bias for all the three different planes. The markerless system always overestimated the measurements compared to the marker-based system when measuring the right hip angle. For the right knee angle the bias was negligible for the measurement in frontal and transverse planes, however, there was a bias between the two methods of measurement for the measurements in the sagittal plane. The mean bias of the markerless system may not be problematic in a practical application as it is always possible to post-process the measurements by the markerless system by this amount. However, the width of the limits of agreement are important in practical situations as there is no way one can offset that for the markerless system.

The width of the agreement indicates how much the measurement produced by the markerless system can deviate from the marker-based system for individual measurements. Based on the width of the limits in the scenarios considered (Fig. 9), it can be concluded that the level of agreement is poor for all the angles measured in the different planes. For low spine angles, the markerless system could produce measurements up to 7${^\circ }$ higher than the marker-bases system in all three planes. For the right hip angle, the deviation could be as high as 12${^\circ }$ in all the planes. For the right knee angle this deviation could be as high as 10${^\circ }$. For lower limb kinematics a measurement of 2${^\circ }$ to 3${^\circ }$ deviation may be acceptable, however, 7${^\circ }$ or more appears to be an unacceptable deviation for the lower limb kinematic measurements given the range of values for this measurement. For this reason it can be concluded that the two measurement systems do not agree when measuring lower limb kinematic measurements during a lunge.

The proposed framework has been applied to the motions generated by a lunge, however, it is flexible enough to model any functional response given the flexibility of the B-spline basis system. In addition to that multivariate functional response (e.g. considering both the angular motion and translation at the same time) can also be incorporated in the modelling framework.

Although the proposed framework can be used to assess agreement between methods of measurement producing functional responses for many different movements, a sensitivity study of the proposed method is needed to find out which components of the modelling framework are most sensitive for the approach.

## Conclusion

In this paper, the agreement between a novel markerless motion capture system and the existing gold standard marker-based system has been assessed for the lower extremity kinematics measurements during a lunge. The assessment of the agreement between the two systems was conducted using the functional responses collected by these two systems in a hierarchical study design. In order to assess the agreement for methods producing functional responses, a novel approach to calculate 95% fLoA using a non-parametric mixed-effects modelling framework has been proposed which allows to calculate 95% fLoA for study designs that an LMM can handle.

Marker-based methods are the gold standard method for measuring three-dimensional kinematics data. A markerless method has been proposed as an convenient and affordable alternative. The level of agreement between the systems, for the angles considered here, was questionable.

It was found that the markerless motion capture system over-estimated kinematic measurement for most angles. There is a substantial bias between the two methods of measurement for most of the angles during a lunge. To use the markerless system for motion capture one must post-process the measurements to remove this bias. The results of this analysis can be used to provide the frame by frame bias adjustment as needed. However, the difference between these two methods of measurement for an individual measurement is substantially higher for all the angles measured. From this, it can be concluded that the markerless cannot be used instead of marker-based motion capture system, in particular when the angle in question represents an internal/external rotation.

### Supplementary Information

Supplementary Information.

## Data Availability

The data are not been publicly available as it is owned by a professional football club. The corresponding author should be contacted to request the data from the study.
