# Supplementary material for: Comparison of markerless and marker-based motion capture systems using 95% functional limits of agreement in a linear mixed-effects modelling framework
Source: Sci Rep. 2023 Dec 18;13:22880. doi: 10.1038/s41598-023-49360-2 (PMC10739832; doi:10.1038/s41598-023-49360-2)
Supplement: Supplementary file 1 — Supplementary Information. [file 41598_2023_49360_MOESM1_ESM.pdf]

Appendix

Different Error Structures for Linear Mixed-effects Model

A linear mixed-effects model often assumes that the within-group error is white noise. However, it may not be a suitable assumption to consider in many situations. In those situations, the within-group error could be *correlated* or *heteroscedastic* or both correlated and heteroscedastic. This correlated and heteroscedastic error is modelled using the covariance matrix $\boldsymbol{\Sigma}$. This is done using the decomposition of the matrix $\boldsymbol{\Sigma}$ into a product of matrices (Pinheiro and Bates, 2000)

$$\boldsymbol{\Sigma} = \boldsymbol{VCV} \tag{1}$$

where, $\boldsymbol{V}$ is a diagonal matrix and $\boldsymbol{C}$ is the correlation matrix. To uniquely identify $\boldsymbol{\Sigma}$ all the diagonal elements of $\boldsymbol{V}$ must be positive (Pinheiro and Bates, 2000).

For a single level LMM, it can be shown that

$$\text{Var}(\epsilon_{ij}) = [V]^2_{jj}, \quad \text{cor}(\epsilon_{ij}, \epsilon_{ik}) = [C]_{jk} \tag{2}$$

where ϵ_{ij} is the error of the jth measurement from the ith subject and ϵ_{ik} is the error for the kth measurement from the same subject. This decomposition of the covariance structure $\boldsymbol{\Sigma}$ into a *variance structure* $\boldsymbol{V}$ and a *correlation structure* $\boldsymbol{C}$ is useful both theoretically and computationally. It allows one to model the two structures separately and then combine them in a more flexible framework. The variance functions for variance component $\boldsymbol{V}$ and correlation structure for the correlation component $\boldsymbol{C}$ will now be described.

Variance functions are used to model different variance structures which can address the issue of heteroscedasticity. The general variance structure for the single level LMM can be defined using variance functions as follows (Pinheiro and Bates, 2000):

$$\text{Var}(\epsilon_{ij}|\boldsymbol{b_i}) = \sigma^2 g^2(\mu_{ij}, \boldsymbol{v}_{ij}, \boldsymbol{\delta}) \tag{3}$$

where, $\mu_{ij} = \text{E}(y_{ij}|\boldsymbol{b_i}), \boldsymbol{v}_{ij}$ is the vector of variance covariates, $\boldsymbol{\delta}$ is the vector of variance parameters,

Table 1: Different variance functions in `nlme` package in R

Function name in `nlme`	description	Var (ϵ_{ij})	$g(.)$
`varFixed`	fixed variance	$\sigma^2 \texttt{time}_{\texttt{ij}}$	$\sqrt{\texttt{time}_{\texttt{ij}}}$
`varIdent`	variances per stratum	$\sigma^2 \delta^2_{s_{ij}}$	$\delta_{s_{ij}}$
`varPower`	power of covariate	$\sigma^2 \lvert\texttt{time}_{\texttt{ij}}\rvert^{2\delta}$	$\lvert\texttt{time}_{\texttt{ij}}\rvert^{\delta}$
`varExp`	exponential of covariate	$\sigma^2 \exp\left(2\,\delta\,\texttt{time}_{\texttt{ij}}\right)$	$\exp\left(\delta\,\texttt{time}_{\texttt{ij}}\right)$
`varConstPower`	constant plus `varPower`	$\sigma^2(\delta_1 + \lvert\texttt{time}_{\texttt{ij}}\rvert^{\delta_2})^2$	$\delta_1 + \lvert\texttt{time}_{\texttt{ij}}\rvert^{\delta_2}$

and $g(.)$ is the variance function assumed to be continuous over $\boldsymbol{\delta}$. The choice of the function $g(.)$ depends on the context. If it is believed that the variance of the within-group error increases linearly with `time` then the variance model would be

$$\text{Var}(\epsilon_{ij}) = \sigma^2 \texttt{time}_{\texttt{ij}} \tag{4}$$

and the corresponding variance function is

$$g(\texttt{time}_{\texttt{ij}}) = \sqrt{\texttt{time}_{\texttt{ij}}}. \tag{5}$$

There are different variance functions and correlation structures available in the `nlme` package in R (R Core Team, 2021; Pinheiro and Bates, 2000). This makes variance functions and correlation structures easily accessible when fitting a mixed-effects model in R. Table 1 lists the different variance functions available in the `nlme` package in R.

In the mixed-effects modelling framework, the correlation structure is used to model the dependency of the within-group error. Historically, these dependency structures have been developed in two areas of statistics: time series analysis and spatial data analysis. The difference between these two types of data is that response variable in time series is usually indexed by one variable (i.e. time) whereas in spatial data, the response is usually indexed by two coordinates of a spatial plane. As the motion capture study involves a time series type response, dependency structures needed for time series analyses will be considered.

To develop a general structure similar to the variance function, consider the case where the dependency of the within-group errors only depend on some position vector $\boldsymbol{p_{ij}}$. In the situation where a univariate response is considered, this position vector is just a scalar. In the case of spatial data analysis for example, this position vector may contain a multidimensional vector. It is also assumed that the correlation structure is *isotropic*. This means the correlation between two errors depends only through some distance, say $d(\boldsymbol{p_{ij}}, \boldsymbol{p_{ik}})$, between these two positional vectors relating to those errors.

A general correlation structure for errors is therefore as follows (Pinheiro and Bates, 2000):

$$\text{cor}(\epsilon_{ij}, \epsilon_{ik}) = h[d(\boldsymbol{p_{ij}}, \boldsymbol{p_{ik}}), \boldsymbol{\rho}], \tag{6}$$

where, $\boldsymbol{\rho}$ is the vector of correlation parameters and $h(.)$ is the correlation function.

This is a very general structure for modelling the dependency in the within-group errors both for the spatial data and time series data. Since the nature of the data in this thesis is similar to time series data, from now on the focus will be on correlation structures relevant for time series data. The correlation in time series data is known as *serial correlation.* Since only time series data are considered, $\boldsymbol{p_{ij}}$ will only contain a scalar position index which will be denoted as p_{ij} since it is no longer a vector. The isotropic assumption will further be simplified to a situation where the correlation only depends on the absolute value of the difference between two position indexes. The general serial correlation structure can now be modelled as (Pinheiro and Bates, 2000)

$$\text{cor}(\epsilon_{ij}, \epsilon_{ik}) = h[\,|p_{ij} - p_{ik}|, \boldsymbol{\rho}] \tag{7}$$

In time series data, the correlation function $h(.)$ is referred to as *autocorrelation* function. A non-parametric estimate of the autocorrelation function is known as the *empirical autocorrelation* function and can be used to examine the serial correlation in the data. Let $r_{ij} = (y_{ij} - \hat{y}_{ij})/\hat{\sigma}_{ij}$ denote the standardised residual from a fitted mixed-effect model where $\hat{\sigma}^2_{ij}$ is the estimate of $\text{Var}(\epsilon_{ij}) = \sigma^2_{ij}$, then the empirical autocorrelation function at lag l is defined as (Pinheiro and Bates, 2000)

$$\hat{\rho}(l) = \frac{\sum_{i=1}^{n} \sum_{j=1}^{n_i - l} r_{ij} r_{i(j+l)}/N(l)}{\sum_{i=1}^{n} \sum_{j=1}^{n_i} r^2_{ij}/N(0)} \tag{8}$$

where, n is the total number of subjects, n_i is the number of observations for the ith subject, $N(l)$ is the number of residual pairs used pairs used in the summation to define the numerator of $\hat{\rho}(l)$, and $N(0)$ is the total number of residuals.

The simplest serial correlation structure is *compound symmetry*, which can be defined as follows:

$$\text{cor}(\epsilon_{ij}, \epsilon_{ik}) = \rho, \quad \forall j \neq k \tag{9}$$

In this situation the autocorrelation function is $h(l, \rho) = \rho$, where $l = |j - k|$. This is a very simplistic correlation structure and might not be very useful in general. The other extreme is the general correlation structure, where the autocorrelation function is

$$h(l, \boldsymbol{\rho}) = \rho_l.$$

This may also not be a useful correlation structure as it requires many correlation parameters to be

estimated. Therefore, this may be useful only to find a more parsimonious correlation structure for exploratory purposes.

The correlation structure that is most relevant for this thesis comes from a different class of linear stationary models: *autoregressive* models and *moving average* models. These models assume that the measurements were taken at discrete time points. Consider ϵ_t as the measurement at time point t. The distance, or *lag*, between two measurements ϵ_t and ϵ_s is $|t - s|$ where lag-1 means the measurements are one unit apart. The autoregressive model assumes that the measurement at the current time is linearly dependent upon the previous measurements plus homoscedastic white noise, a_t, centred at zero, $\mathrm{E}(a_t) = 0$ (Pinheiro and Bates, 2000).

$$\epsilon_t = \phi_1 \epsilon_{t-1} + \ldots + \phi_p \epsilon_{t-p} + a_t \tag{10}$$

The number of previous observations on which the current measurement depend upon is called the *order* of the autoregressive model. The order of the autoregressive model here is p and the model is denoted as an AR(p) model. Note that p is used previously to denote the number of fixed-effects in a LMM. For this section, p will be used as the order of an AR model. The model also contains the same number of correlation parameters, $\boldsymbol{\phi} = (\phi_1, \ldots, \phi_p)'$ as the order of the model. The correlation function for an AR(1) model is follows (Pinheiro and Bates, 2000):

$$h(k, \phi) = \phi^k, \quad k = 0, 1, 2, \ldots \tag{11}$$

where k is the distance between two time points. The correlation function beyond the AR(1) model does not have any simple representation. It is defined recursively through the difference equation (Pinheiro and Bates, 2000)

$$h(k, \phi) = \phi_1 h(|k-1|, \boldsymbol{\phi}) + \ldots + \phi_p h(|k-p|, \boldsymbol{\phi}), \quad k = 1, 2, 3, \ldots \tag{12}$$

Moving average models assume that the current observations are a linear combination of independent and identically distributed white noise terms (Pinheiro and Bates, 2000)

$$\epsilon_t = \theta_1 a_{t-1} + \ldots + \theta_q a_{t-q} + a_t. \tag{13}$$

The number of white noise terms with lag, q, is the order of the moving average model which is denoted by MA(q) model. Note that q is used previously to denote the number of random-effects in a LMM. For this section, q will be used as the order of a MA model. There are q correlation parameters in this model $\boldsymbol{\theta} = (\theta_1, \ldots, \theta_q)'$.

The correlation function for an MA(q) model for the observations with k distance apart is as follows

Table 2: Name of the correlation functions available in the `nlme` package in R.

Name of correlation function in `nlme`	description
`corCompSymm`	compound symmetry
`corSymm`	general
`corAR1`	autoregressive of order 1
`corARMA`	autoregressive-moving average
`corExp`	exponential
`corGaus`	Gaussian
`corLin`	linear
`corRatio`	rational quadratic
`corSpher`	spherical

(Pinheiro and Bates, 2000):

$$h(k, \boldsymbol{\theta}) = \begin{cases} \frac{\theta_k + \theta_1\theta_{k-1} + \ldots + \theta_{k-q}\theta_q}{1+\theta_1^2+\theta_2^2+\ldots+\theta_q^2}, & k = 1, 2, \ldots, q, \\ 0, & k = q+1, q+2, \ldots \end{cases} \tag{14}$$

A combination of an autoregressive and a moving average model is called an autoregressive-moving average model and denoted by ARMA (p, q) with the order p for the autoregressive model and order q for the moving average model. This model can be written as follows (Pinheiro and Bates, 2000):

$$\epsilon_t = \sum_{i=1}^{p} \phi_i \epsilon_{t-i} + \sum_{j=1}^{q} \theta_j a_{t-j} + a_t \tag{15}$$

The correlation function for this model is defined recursively as follows (Pinheiro and Bates, 2000):

$$h(k, \boldsymbol{\rho}) = \begin{cases} \phi_1 h(|k-1|, \boldsymbol{\rho}) + \ldots + \phi_p h(|k-p|, \boldsymbol{\rho}) + \\ \quad \theta_1 \psi(k-1, \boldsymbol{\rho}) + \ldots + \theta_q \psi(k-q, \boldsymbol{\rho}), & k = 1, 2, \ldots, q \\ \phi_1 h(|k-1|, \boldsymbol{\rho}) + \ldots + \phi_p h(|k-p|, \boldsymbol{\rho}), & k = q+1, q+2, \ldots, \end{cases} \tag{16}$$

where $\psi(k, \boldsymbol{\rho}) = \mathrm{E}(\epsilon_{t-k} a_t)/\mathrm{Var}(a_t)$. Table 2 lists the available correlation functions in the `nlme` package. Note that this table includes correlation structures for time series data and correlation structures for spatial data. Only the correlation structure for time series data has been discussed in this thesis and the details for the spatial correlation structure can be found in Pinheiro and Bates (2000).

References

J. Pinheiro and D. Bates. *Mixed-effects models in S and S-PLUS*. Springer science & business media, 2000.

R Core Team. *R: A Language and Environment for Statistical Computing.* R Foundation for Statistical Computing, Vienna, Austria, 2021. URL `https://www.R-project.org/`.
